# Human A53T α-Synuclein Causes Reversible Deficits in Mitochondrial Function and Dynamics in Primary Mouse Cortical Neurons

**DOI:** 10.1371/journal.pone.0085815

**Published:** 2013-12-31

**Authors:** Li Li, Sashi Nadanaciva, Zdenek Berger, Wei Shen, Katrina Paumier, Joel Schwartz, Kewa Mou, Paula Loos, Anthony J. Milici, John Dunlop, Warren D. Hirst

**Affiliations:** 1 Neuroscience Research Unit, Pfizer, Inc., Cambridge, Massachusetts, United States of America; 2 Compound Safety Prediction Group, Pfizer, Inc., Groton, Connecticut, United States of America; University of Pittsburgh, United States of America

## Abstract

Parkinson’s disease (PD) is the second most common neurodegenerative disease. A key pathological feature of PD is Lewy bodies, of which the major protein component is α-synuclein (α-syn). Human genetic studies have shown that mutations (A53T, A30P, E46K) and multiplication of the α-syn gene are linked to familial PD. Mice overexpressing the human A53T mutant α-syn gene develop severe movement disorders. However, the molecular mechanisms of α-syn toxicity are not well understood. Recently, mitochondrial dysfunction has been linked with multiple neurodegenerative diseases including Parkinson’s disease. Here we investigated whether mitochondrial motility, dynamics and respiratory function are affected in primary neurons from a mouse model expressing the human A53T mutation. We found that mitochondrial motility was selectively inhibited in A53T neurons while transport of other organelles was not affected. In addition, A53T expressing neurons showed impairment in mitochondrial membrane potential and mitochondrial respiratory function. Furthermore, we found that rapamycin, an autophagy inducer, rescued the decreased mitochondrial mobility. Taken together, these data demonstrate that A53T α-syn impairs mitochondrial function and dynamics and the deficit of mitochondrial transport is reversible, providing further understanding of the disease pathogenesis and a potential therapeutic strategy for PD.

## Introduction

Parkinson’s disease (PD) is the second most common neurodegenerative disease, affecting 1% of the population older than 60 and up to 3% of people older than 85 years [1]. This movement disorder is characterized by resting tremor, rigidity, postural reflex impairment and bradykinesia. Molecular mechanisms of the disease are still unclear. However, previous studies have shown that both environmental and genetic factors play a causal role in PD [2-5].

α-synuclein (α-syn) is the major protein component of Lewy bodies, the pathological hallmark of PD [6]. Human genetic studies have shown that mutations within the α-synuclein gene, A53T, A30P, E46K, and multiplications of this gene are linked to familial PD [7]. More recently, genome-wide association (GWAS) studies have also demonstrated that *SNCA*, which encodes α-syn, is linked to sporadic PD [8]. α-syn is an abundant 140-residue protein, which is primarily found in neural tissues including the cortex, hippocampus, substantia nigra, thalamus, cerebellum and spinal cord [9]. It is localized in the cytosol, nucleus and mitochondria and is enriched presynaptically. Increased expression of α-syn can drive its aggregation, and A53T α-syn has increased propensity to oligomerize [10] and aggregate into fibrillar forms [11,12]. Mice expressing A53T α-syn develop severe motor deficits leading to paralysis and death [13]. These animals also develop age-dependent α-syn inclusions that recapitulate the pathology seen in human PD patients. Although α-syn plays a critical role in the pathogenesis of PD the underlying molecular mechanisms of α-syn toxicity are still unclear.

Mitochondrial dysfunction has been linked with multiple neurodegenerative diseases including PD [14,15]. Recent reports have shown that α-syn exists in mitochondria and can affect mitochondrial function [16,17]. For example, overexpression of A53T α-syn was shown to inhibit Complex I activity in the dopaminergic neurons of transgenic mice [18], depolarize mitochondrial membrane potential and increase reactive oxygen species in human neuroblastoma cells [19], and induce mitochondrial autophagy in neurons expressing the A53T mutation [18,20]. In addition, it was recently shown that α-syn affects mitochondrial motility [21]. In the current study, we investigated whether the human A53T α-syn mutation expressed in primary cortical neurons from mice affects mitochondrial transport, membrane potential and respiratory function and found that all these parameters were impaired in the presence of the mutant α-syn. We also investigated whether the defective mitochondrial phenotype is reversible and demonstrated that rapamycin rescued the mitochondrial mobility in the A53T α-syn neurons.

## Materials and Methods

### Plasmids and reagents

Mitochondria, autophagosomes and rapidly-moving small vesicles were labeled with pDsRed2 –mito (Clontech), EGFP-LC3 and mCherry-synaptophysin, respectively. TMRM, MitoTracker Red and all culture media except for glucose and NaCl were from Life Technologies (Carlsbad, CA). Rapamycin was purchased from Sigma (St. Louis, MO). 

### Cortical neuron culture

All mouse procedures were approved by Pfizer, Inc and were in accordance with Pfizer’s Internal Animal Care and Use Committee guidelines and the National Institutes of Health Guide for the Care and Use of Laboratory Animals. A53T α-syn mice and their wild-type littermates were ordered from the Jackson Laboratory (Bar Harbor, ME). Homozygous transgenic C57BI/C3H mice were generated expressing human A53T α-syn under the control of the prion promoter. The generation and phenotype of these mice has been previously described [13]. Cortical (E16) neuron cultures were prepared as described by Banker and Goslin (1998) [22]. Briefly, cortices were dissected, trypsinized and dissociated. Dissociated cortical neurons were plated on 0.5mg/mL poly-D-lysine (Sigma)-coated glass bottom dishes (MatTek). In some experiments, dissociated neurons were resuspended in Nucleofector solution (Mouse neuron kit, Lonza) and transfected with an Amaxa Nucleofector following the manufacturer’s directions. Fluorescence was seen in 48 hours. Neurons were plated in Neurobasal medium supplemented with B27 supplement, 2mM glutamine, 16mM glucose (Sigma) and 37.5mM NaCl (Sigma). 

### Live-cell confocal imaging

The spinning disk confocal microscope consisted of a Nikon Eclipse Ti (Nikon) base with spinning disk CSU-X1 (Yokogawa), Nikon 100X/1.40NA, Nikon 60X/1.40NA or Nikon 40X/1.30NA objectives, a Nikon perfect focus system for continuous automatic focusing of the sample during time-lapse imaging and an EMCCD camera (Andor iXON X3). During time-lapse microscopy neurons were kept at 37 °C within an incubation chamber (Okolab). All images were collected, measured and compiled in ImageJ (NIH) or MetaMorph imaging software (Molecular Devices). Figures were compiled in Illustrator (Adobe). A Zeiss LSM510 confocal microscope was also used to acquire some time-lapse images at the beginning of the project.

### Quantitative analysis of mitochondrial transport and morphology

Axonal processes were chosen for mitochondrial transport analysis based on known morphological characteristics [23-26]. A process was considered an axon if it was at least twice the length of any of other processes, with thin and uniform diameter, sparse branching and prominent growth cones. In addition, axonal mitochondria are more sparsely distributed along axons. Mitochondria without fission and fusion were selected for the measurement of mitochondria transport. All mitochondrial transport and shape/morphology measurements were made with the aid of MetaMorph software (Molecular Devices) and ImageJ (NIH), respectively. Live-cell images were collected with 40X or 60X or 100X objectives. All images used for quantification of mitochondrial transport were collected at 3-60s intervals for a period of 5-20min. To represent, in a single image, the velocity of mitochondrial transport over the imaging period, we compiled kymographs. These kymographs consisted of taking the maximum pixel value from a five pixel-wide line drawn along axons. The maximum value within a five pixel-wide line (instead of using a single pixel-wide line) was used to assure that we recorded all mitochondria transport along axons. In kymographs, bright vertical lines represented stationary mitochondria and mobile mitochondria were represented by diagonal lines. To quantify mitochondrial morphology, the mitochondrial morphology macro developed by Dr. Charleen Chu’s lab in the University of Pittsburg was adapted [27]. Briefly, the fluorescent images of cortical neurons transfected with DsRed-mito or loaded with MitoTracker Red were threshold first to highlight mitochondria and the Shape Descriptors plug-in in ImageJ was used to measure parameters of mitochondria morphology, including mitochondria length (defined by the length of major axis) [28], mitochondria width (defined by the length of minor axis), circularity (4π*area/perimeter^2), aspect ratio (major_axis/minor_axis) and roundness (4*area/(π*major_axis^2)). Circularity, roundness and aspect ratio describe the shape of mitochondria. A circularity value of 1 indicates a perfect circle. As the circularity value approaches 0, it indicates an increasingly elongated shape. Only mitochondria which were not touching other mitochondria were included in the analysis.

### Assessment of mitochondrial membrane potential (ψ_m_)

To monitor the mitochondrial membrane potential in wt and A53T neurons, primary cortical cultures were treated with 10nM MitoTracker Red CMXRos (Invitrogen) for 30min, washed three times with pre-warmed culture media as reported previously [29] and imaged on Nikon spinning-disk confocal microscope in an incubation chamber (37°C, 5% CO2) to keep cells live and healthy. 30-40 fields were randomly selected and imaged in each condition. The cytosol area in the soma of all the neurons was selected and the average fluorescence intensity was measured with the aid of Metamorph (Molecular devices) or ImageJ (NIH). Student’s t test was performed to compare MitoTracker labeling in wt vs. A53T neurons. To confirm the MitoTracker Red data, TMRM (Invitrogen) was used to measure mitochondria membrane potential. In brief, cortical neurons were loaded with 10nM TMRM for 30min and imaged with 10nM TMRM in the media to maintain the equilibrium distribution of fluorophore [30]. To assess the effects of rapamycin on the mitochondrial membrane potential, cortical neurons from wt and A53T mice were plated onto poly-D-lysine coated 96-well microplates (Greiner Bio-one). At 6DIV, cultures were treated with rapamycin (1μM) for 7 hours. 30min prior to the imaging, MitoTracker (25nM) Red and NucBlue Live Ready Probes Reagent (1:1000, Life Technologies) were loaded into cultures. Neurons were then washed and imaged live using the Cellomics Arrayscan High Contact Analysis Reader (Thermo Scientific) at 37°C and 5% CO2. Widefield images were taken using a 40X objective, scanning 64 fields per well. Image Analysis was performed with the Cellomics Arrayscan VTI to quantify the intensity of Mitochondria Red within the neuronal cell bodies. 

### Seahorse extracellular flux assay

Primary cortical neurons were cultured on XF96 plates (Seahorse Bioscience, Billerica, MA) at a density of 40,000 cells/well. The neurons were grown in neurobasal medium supplemented with B27, glutamine, glucose and NaCl for 7-14 days. On the day of the assay, the cell culture medium was replaced with 150μL/well of pre-warmed low-buffered medium (DMEM base medium supplemented with 25 mM glucose, 1 mM sodium pyruvate, 31 mM NaCl, 2 mM glutamine, pH 7.4) and the cells incubated at 37°C for 30 min in a non-CO_2_ incubator. Injection reagents were prepared in DMSO and then diluted to the appropriate concentrations in the low-buffered medium. Oxygen consumption rates (OCR) and extracellular acidification rates (ECAR) of the neurons were measured at 37°C using a Seahorse XF96 Extracellular Flux Analyzer (Seahorse Bioscience). Four baseline measurements of OCR and ECAR were taken before injection of mitochondrial modulators. The modulators used were rotenone (1 μM), antimycin (1 μM), oligomycin (3 μM), and FCCP (3 μM). Three or four readings were taken after each addition of the mitochondrial modulator. Basal OCR and ECAR as well as the percentage changes in OCR and ECAR upon addition of the mitochondrial modulators were recorded and calculated by the XF-96 software. After the OCR and ECAR measurements, protein measurements from each well were taken using the BCA assay (Pierce, Rockford, IL) in order to normalize basal OCR to protein content. 

### Statistical analysis

All statistical tests and graphs were performed with GraphPad Prism (La Jolla, CA). 

## Results

### Mitochondrial transport was inhibited in A53T α-syn axons

**Figure 1:**
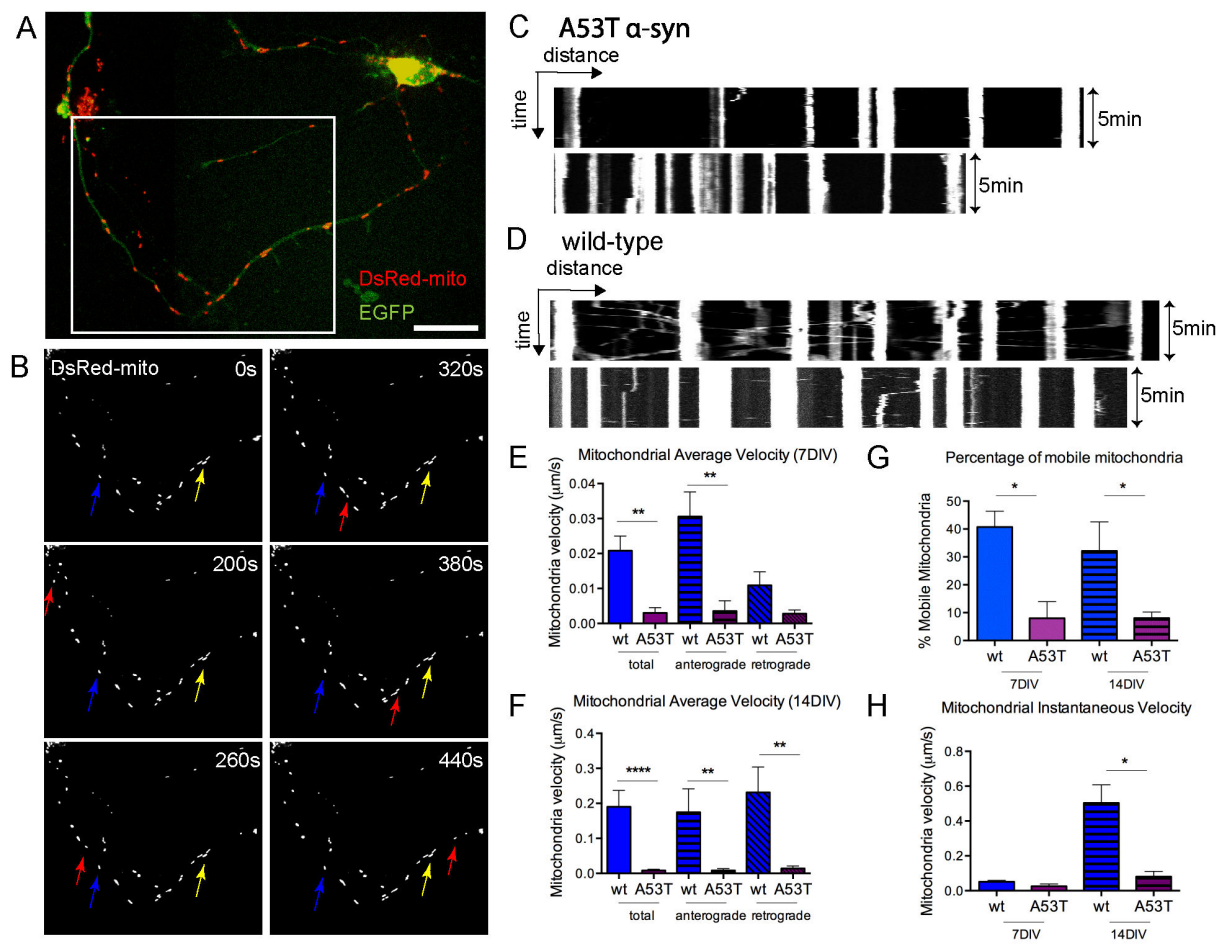
Mitochondrial transport was reduced in A53T α-syn axons. **A**, An example of a cortical neuron from A53T mice transfected with dsRed-mito (red) and EGFP (green) at the time of plating. **B**, Montage of images (~7min) at the dsRed-mito channel from a 20min confocal time-lapse of the neurite area (outlined by white box in A). In the image area, the majority of mitochondria were stationary indicated by the blue and yellow arrows. Red arrows pointed to a mobile mitochondrion that entered the area at ~200s and moved along the axon. **C**, **D**, Kymographs constructed from five-pixel wide lines drawn through the extent of axons from wt or A53T neurons. Fluorescence intensities along kymograph lines are plotted horizontally for each frame of the 5min time-lapse with time interval of 3s (101 frames). Time is indicated on the y-axis and distance is indicated on the x-axis. In A53T neurons (**C**), most of mitochondria are stationary represented by the bright vertical lines. In wt neurons (**D**), there are diagonal lines representing moving mitochondria. **E**, **F**, Bar graphs from one representative experiment at 7DIV (**E**) and 14DIV (**F**) showing that mitochondria transport velocity was reduced significantly in A53T neurons. In each condition, 53-145 mitochondria were analyzed and ≥ 4 independent experiments showed consistent results. **G**, Percentage of mobile mitochondria significantly decreased in A53T (~8%) compared to that in wt neurons (>30%). Mobile mitochondria are defined as mitochondria that moved at ≥0.005 μm/s. H, Mitochondrial instantaneous velocity also reduced in A53T axons. Data plotted as Mean +/- SEM. t test, *p<0.05; **p<0.01; ****p<0.0001. Scale bar=20μm. wt=wild type, A53T=A53T α-synuclein, μm=micrometers, s=seconds, min=minutes, DIV=days *in* *vitro*.

We initially sought to investigate whether human α-syn mutation (A53T) affects mitochondrial mobility in primary neurons. Previously published data has shown that the transgenic mouse line expressing A53T human α-syn in CNS (A53T α-syn mice) recapitulates features of human PD patients including motor impairment and age-dependent intra-neuronal α-syn inclusions [13]. However, the molecular mechanism of A53T α-syn toxicity is not clearly understood. We used primary cortical cultures from the A53T α-syn mice and robust expression of human α-syn was observed in these cultures; the majority of neurons in the A53T cultures expressed human α-syn (Figure S1). Mitochondria were visualized either by use of MitoTracker Red CMXROS (not shown) or by transfection of DsRed-mito (Figure 1A) into cortical neurons derived from A53T α-syn mice or their wild-type (wt) littermates (E16). EGFP was co-transfected into neurons to label the morphology of neurons (Figure 1A). Confocal time-lapse live images were acquired at the age of 6-7DIV (days in vitro) and 14DIV at intervals of 3-20s for 5-20min to capture mitochondrial mobility along neurites. Only axons were selected for the quantification of mitochondrial mobility.

Representative images and kymographs are shown in Figure 1A-D. As demonstrated previously [31], mitochondria are either stationary or mobile along the neurite (Figure 1B-D, Movie S1,S2). Overall transport of mitochondria in wt vs. A53T was analyzed by calculating the distance between the position of individual mitochondria at the start and end of time-lapse recordings and dividing by the time elapsed. This yielded an overall velocity of transport for each mitochondrion that includes mobile and stationary periods. In A53T neurons, the average velocity of mitochondria transport was 0.003±0.001μm/s (n=53) compared to 0.021±0.004μm/s in wt (n=94) at 7DIV (Figure 1E). In the more mature neurons, 14DIV, there was a further increase in the average velocity of mitochondrial mobility to 0.190±0.047μm/s (n=85) and the effects of the A53T were more pronounced, reducing the velocity to 0.008±0.004μm /s (n=107) (Figure 1F). In addition to overall mitochondrial average velocity, the anterograde and retrograde velocity was reduced (Figure 1E, 1F). Thus in both young (7DIV) and mature (14DIV) A53T neurons mitochondria mobility was impaired. Mitochondria were subsequently classified as mobile (velocity>=0.005 μm/s) or stationary (velocity<0.005 μm/s) [26,32]. Consistent with reduction of transport velocity in A53T neurons, the percentage of mobile mitochondria in A53T derived primary neurons was reduced by >70% in both young and mature neurons (Figure 1G). Importantly the fraction of mobile mitochondria (>30%) in wild type neurons was consistent with values determined for axons of cortical [24] and hippocampal [32-36] neurons. In addition, we measured instantaneous speed of mitochondria by only including mobile mitochondria [26,37,38]. The mitochondrial instantaneous velocity in wt neurons averaged at 0.051±0.009μm/s and 0.504±0.104μm/s at 7 and 14DIV respectively similar as previously reported [31,39], and the instantaneous velocity decreased in A53T neurons at both ages (Figure 1H). Overall the results suggested A53T α-syn inhibited mitochondria transport through reducing fraction of mobile mitochondria and mitochondrial velocity.

### Transport velocity of other organelles was not inhibited in A53T α-syn neurites

**Figure 2:**
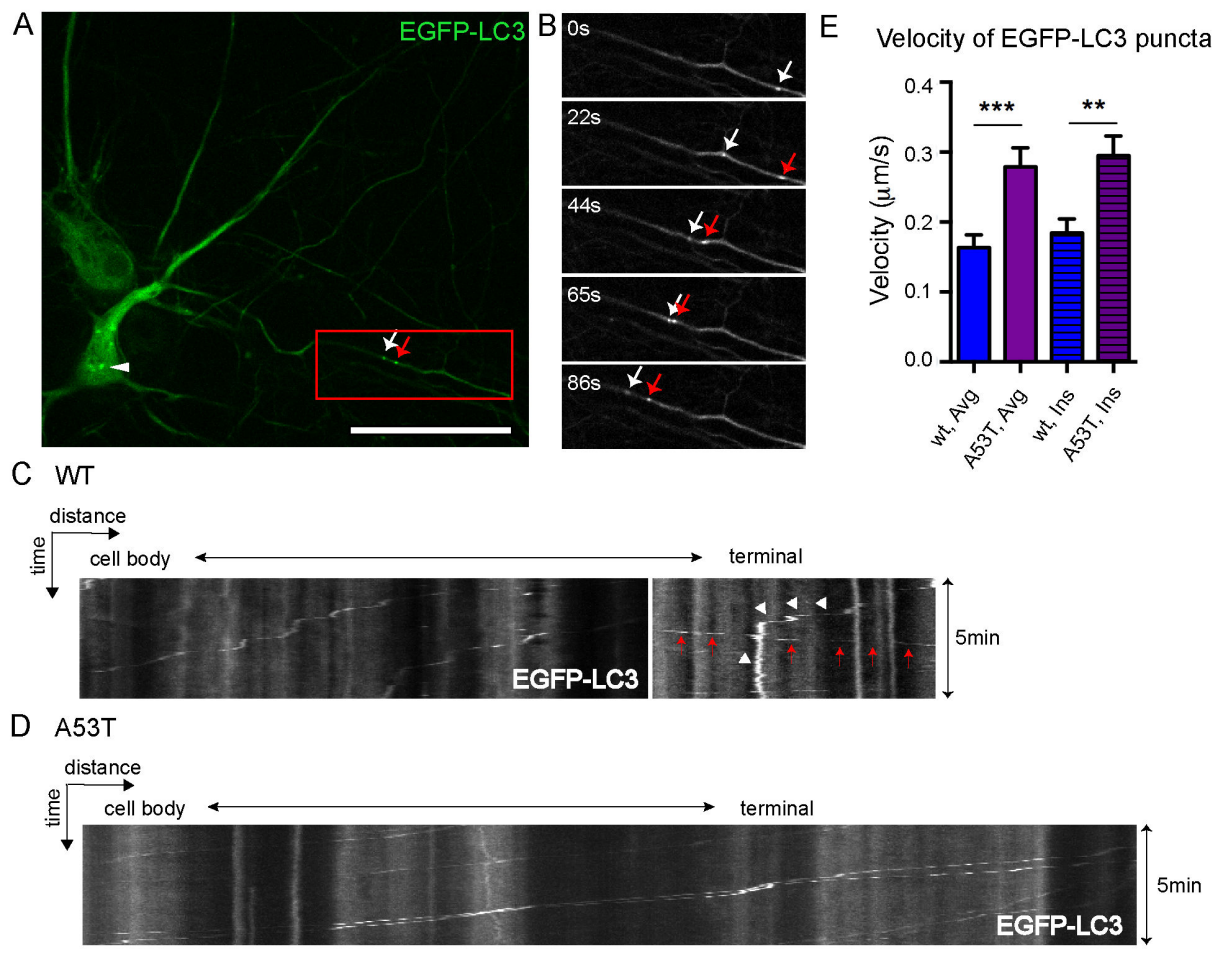
Transport velocity of autophagosomes was not substantially reduced in A53T α-syn neurons. **A**, An A53T α-syn cortical neuron transfected with EGFP-LC3 (green) showing diffuse signal in the cytosol and puncta which are autophagosomes. Autophagosomes were visible at the soma (white arrowhead) and a neurite (white and red arrows). **B**, Montage of images (~1min) from a 5min confocal time-lapse of the neurite area (outlined by red box in A). Two autophagosomes (white and red arrows) moved retrogradely along the neurite. **C**,**D**, Representative kymographs of autophagosome mobility demonstrated that most of autophagosomes in wt and A53T neurons underwent active retrograde movement, however, one of them in the wt group (white arrowhead) paused at the end of the imaging period and another one (red arrows) moved anterogradely. **E**, Bar graphs demonstrating that the average (Avg.) velocity and the instantaneous (Ins.) velocity of autophagosome movement were not reduced in A53T neurons, however, they almost doubled in the mutant cortical neurons. Data were pooled from 3 independent experiments and 309 autophagosomes were included in the analysis. Data plotted as Mean +/- SEM. t test, **p<0.01; ***p<0.001. Scale bar=20μm. wt=wild type, A53T=A53T α-synuclein, μm=micrometers, s=seconds, min=minutes.

To determine whether human A53T α –syn inhibits all cargo movement, cortical neurons were transfected with EGFP-LC3, which selectively labels autophagosomes [40] or mCherry-synaptophysin, which selectively labels synaptic vesicles [41-43]. In cells EGFP-LC3 accumulates in autophagosomes, which form in the process of autophagy [40]. Consistent with previous studies [44,45], EGFP-LC3-positive dots were detected along the neurites (Figure 2A, arrows) as well as in the soma (Figure 2A, arrowhead). Autophagosomes were identified as compartments with strong EGFP-LC3 and with a diameter of 0.5-1um [45]. The majority of LC3 puncta displayed robust motility and moved retrogradely (Figure 2B, 2C), however, there were few puncta moving anterogradely (Figure 2C, red arrows). Interestingly, the movement of LC3-positive vesicles exhibited a unique progressive pattern (Figure 2C, red arrows) with intermittent pausing (Figure 2C, white arrowheads) whereas mitochondria were frequently stationary (Figure 1D). Despite inhibition of mitochondrial transport, A53T did not reduce the speed of autophagosomes (Figure 2C-E). In contrast the average (Avg.) velocity of autophagosomes was 0.28±0.03 μm/s in A53T neurons compared to 0.16±0.02 μm/s in wt neurons (Figure 2E). The instantaneous (Ins.) velocity was 0.30±0.03 μm/s in A53T neurons compared to 0.18±0.02 μm/s in wt neurons (Figure 2E). We also investigated whether human A53T α –syn inhibits movement of mCherry-synaptophysin-positive vesicles. As shown by others [42], the synaptophysin cargo targets small, rapidly moving particles (Figure S2A, B). The velocity of mCherry-synaptophysin-positive vesicles was not significantly inhibited in A53T neurons (Figure S2C). Overall these studies underscore the specificity of A53T α-syn on mitochondria versus general cargo mobility. 

### Mitochondrial gross morphology was not substantially affected in A53T α-syn cortical neurons

**Figure 3:**
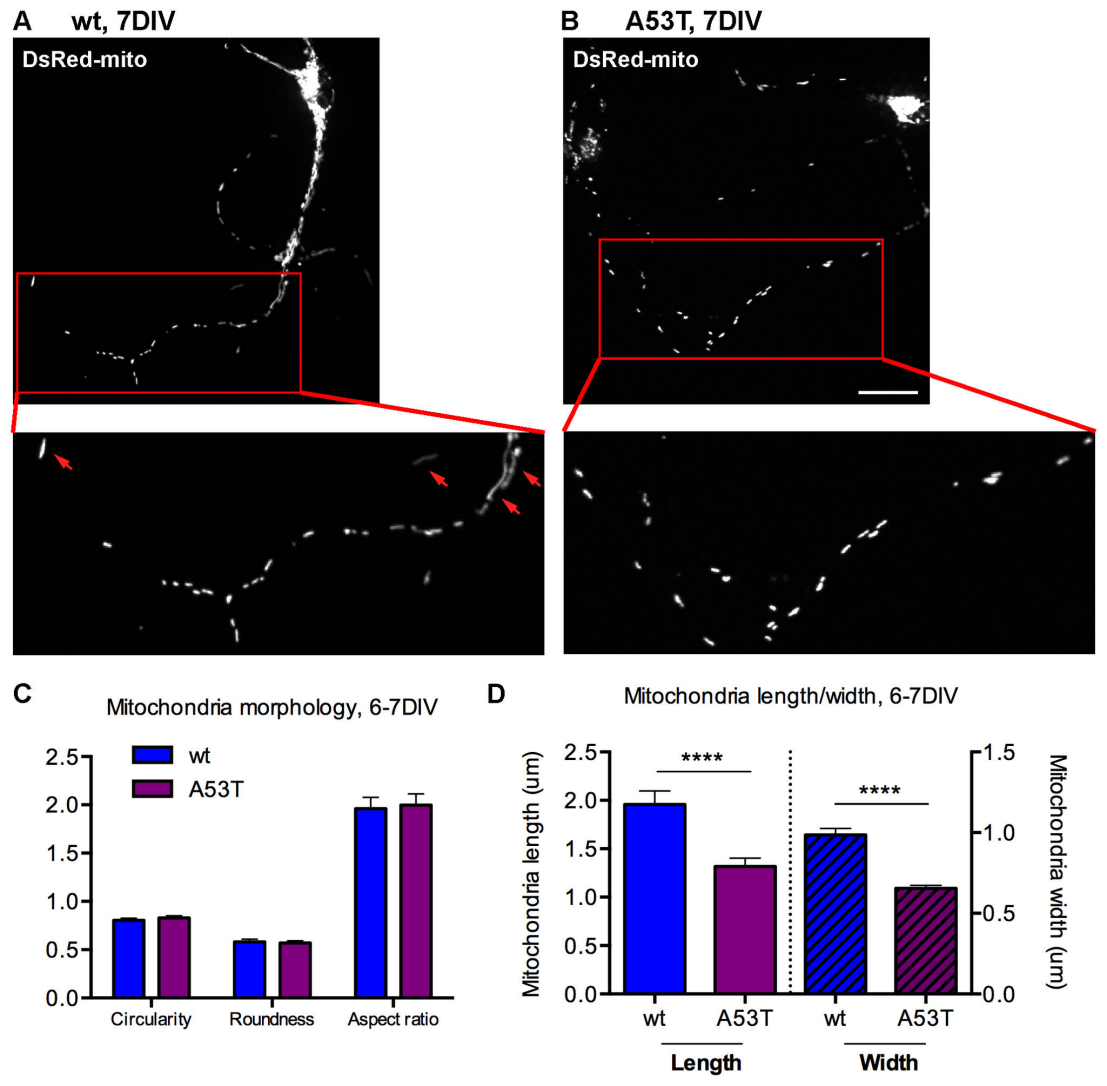
Mitochondrial shape was not substantially affected in A53T cortical neurons but the length was shortened. **A**, **B**, Examples of mitochondrial morphology in wt (**A**) and A53T (**B**) cortical neurons at 7DIV. Cortical neurons were transfected with DsRed-mito. Higher magnification images at the bottom were from the areas highlighted by red boxes at the top images. **C**, Quantification of mitochondrial shape parameters (circularity, roundness and aspect ratio) in wt and A53T neurons at 6-7DIV in one representative experiment. There was no significant change in A53 neurons vs. wt neurons. **D**, Summary of mitochondrial length and mitochondria width in wt and A53T neurons from one representative experiment. Data plotted as Mean +/- SEM. t-test was used (****p<0.0001). Experiments were repeated 3 times (see results in Figure S3 and tables S1 & S2). Scale bar=20μm. wt=wild type, A53T=A53T α-synuclein, μm=micrometers, DIV=days *in* *vitro*.

Previous studies showed that A53T can induce alterations of mitochondrial morphology in SH-SY5Y cells [46], Hela cells [47], the spinal cord and the substantia nigra pars compacta neurons in 12 months but not 6 months [21]. To determine if A53T α-syn can affect mitochondrial gross morphology in cortical neuronal cultures, neurons derived from A53T or wt mice were transfected with DsRed-mito. The mito-morphology macro in Image J [27] was adapted to measure mitochondrial circularity, roundness, aspect ratio and length (details in Materials and Methods). As shown in Figure 3A and 3B, most of mitochondria in both wt and A53T axons displayed as small, vesicular, or short vesicular-tubular structures. However, there were a few elongated, tubular or filamentous structures occupying the wild-type axon (red arrows). At 6-7DIV (Figure 3A-C, table S1) and 13-14DIV (table S2), there was little change of circularity or roundness or aspect ratio of mitochondria in cortical neurons. In agreement with these observations, an independent study reported 6 or 12-month-old mice did not exhibit any significant changes of mitochondrial morphology in the cortex [21]. Interestingly, mitochondria length was shorter at 6-7DIV in A53T neurons (Figure 3D, left part; table S1). The average length of mitochondria reduced significantly from 1.96±0.14μm in wt neurons to 1.32±0.08μm in A53T neurons (Figure 3D, left part) and the results in three other independent experiments showed the same trend (Figure S3A; table S1). The findings recapitulated the results reported previously [48]. With unchanged aspect ratio (aspect ratio=major_axis/minor_axis), shorter mitochondria length in A53T neurons suggested shorter mitochondrial width and it is consistent with the results (Figure 3D, right part; Figure S3B). However, mitochondrial length was unchanged in A53T neurons at 13-14DIV (Figure S3C). MitoTracker Red was also used to measure mitochondrial morphology and the results are consistent with DsRed-mito results (Experiment 4 in table S1, Experiment 2& 3 in table S2). Taken together, these data demonstrate that A53T α-syn did not affect mitochondria shape but shortened mitochondria length and width only at 6-7DIV.

### Mitochondrial membrane potential was depolarized in A53T α-syn neurons

**Figure 4:**
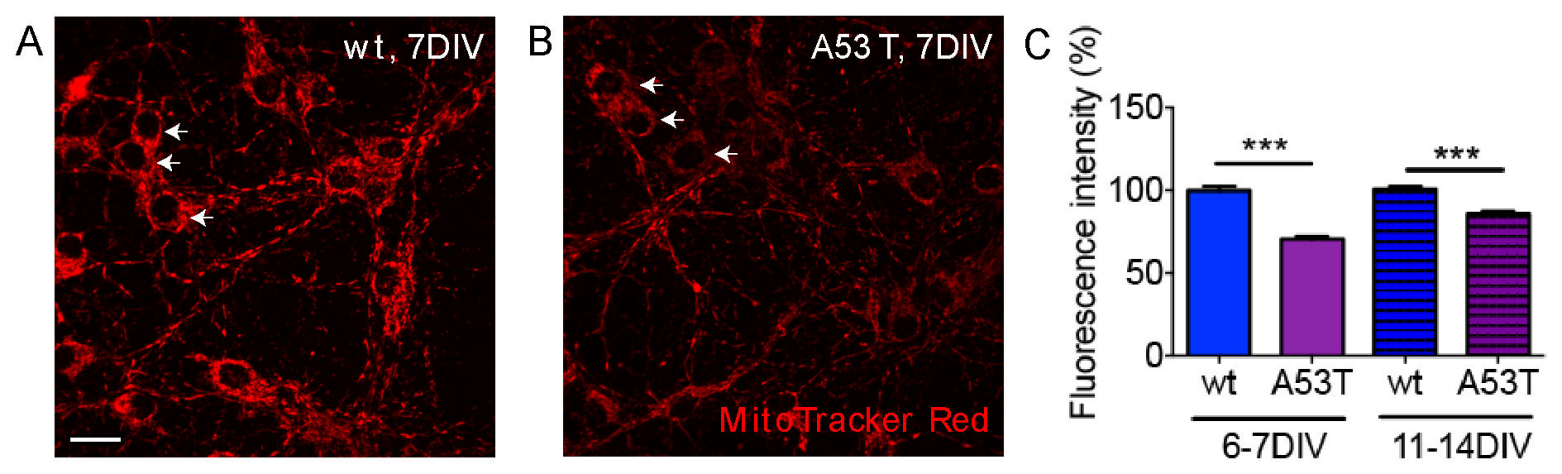
A53T α-syn induced loss of mitochondrial membrane potential. **A**, **B**, Representative images of mitochondria stained with MitoTracker Red. White arrows point to a few cell bodies and only the cytosol area was stained with MitoTracker red. Fluorescence intensity of MitoTracker Red was lower in A53T neurons compared to that in wt neurons. **C**, Bar graphs show a significant reduction in MitoTracker Red fluorescence intensity (normalized) in A53T neurons compared to wt neurons in 6-7 DIV and 11-14DIV. Since accumulation of MitoTracker Red is dependent on mitochondrial transmembrane potential, it suggests a loss of mitochondrial membrane potential in A53T neurons. Data plotted as Mean +/- SEM. Data were pooled from 3 independent experiments and > 150 neurons were measured in each condition (t-test, ***p<0.001). Scale bar=20μm. wt=wild type, A53T=A53T α-synuclein, DIV=days *in* *vitro*.

We next investigated the effects of A53T α-syn on mitochondrial membrane potential (ψ_m_). Primary cortical neurons of each strain were stained with the ψ_m_-sensitive probe, MitoTracker Red [29,49] as described in the Materials and Methods. We used a very low concentration (10nM) of MitoTracker Red to minimize any adverse effects on mitochondria [50,51]. The fluorescence intensity of MitoTracker Red was lower in A53T neurons (Figure 4B) than in wt neurons (Figure 4A). Pooled data from 3 independent experiments demonstrated that ψ_m_ was significantly reduced in A53T α-syn neurons at both 6-7DIV and 11-14DIV (Figure 4C). In each condition, at least 150 neurons were analyzed for the average fluorescence intensity of the indicator. Similar results were obtained with another mitochondrial membrane potential-sensitive dye, TMRM (Figure S4). Thus, human A53T α-syn caused a decrease in ψ_m_ in cortical neurons. 

### Mitochondrial respiration and the maximum respiratory capacity were decreased in A53T α-syn neurons

**Figure 5:**
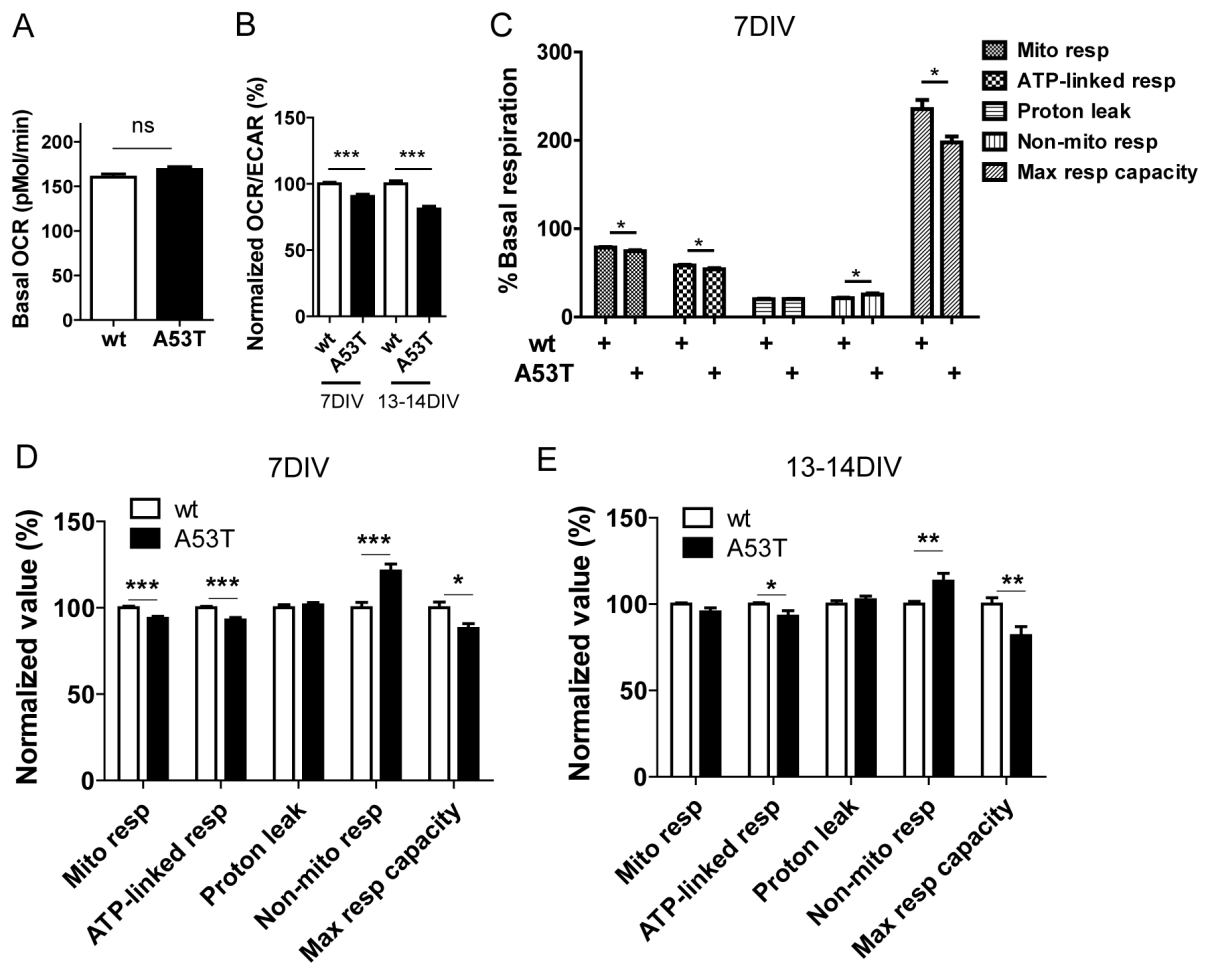
Overexpression of human A53T α-syn impaired mitochondrial respiration and the maximum respiratory capacity. Bioenergetic parameters of A53T and wt neurons were analyzed using a Seahorse XF96 analyzer as described in Materials and Methods. **A**, Basal OCR values were similar in wt and A53T neurons. **B**, A53T neurons had a lower OCR/ECAR ratio than wt both at 7DIV and at 13-14 DIV. **C**-**E**, A53T neurons had lower mitochondrial respiration, lower ATP linked respiration, higher non-mitochondrial respiration and lower maximum respiratory capacity than wt neurons at 7DIV (**C**,**D**) and at 13-14 DIV (**E**). Representative data from one experiment for neurons at 7DIV are shown as in C. Pooled data from 3 independent experiments for neurons at 7DIV are shown in D. As in **E**, pooled data from 3 independent experiments for neurons at 13-14 DIV are shown. Data plotted as Mean +/- SEM (t-test, *p<0.05; **p<0.01; ***p<0.001; ns=non-significant). wt=wild type, A53T=A53T α-synuclein, pMol=picomole, min=minutes, DIV=days *in* *vitro*, OCR=oxygen consumption rates, ECAR=extracellular acidification rates.

The primary physiological function of mitochondria in cells is the generation of ATP by oxidative phosphorylation. In order to determine the effect of the A53T α-syn mutation on cellular respiration, oxygen consumption rates (OCR) and extracellular acidification rates (ECAR) of A53T neurons and wt neurons were measured in an XF96 extracellular flux analyzer as described in Methods and Materials. There was no significant difference between the basal oxygen consumption rate of A53T neurons (169.1±3.0 pmol/min/40,000 cells) and that of wild type neurons (160.3±3.6pmol/min/40,000 cells) (Figure 5A). However, the OCR/ECAR ratio of A53T neurons was lower (p< 0.05) than that of wt neurons both at 7 DIV and at 13-14 DIV (Figure 5B), indicating the A53T neurons were more glycolytic than the wt neurons. 

We next measured mitochondrial respiration, ATP-linked respiration, proton leak, non-mitochondrial respiration, and maximum respiratory capacity of neurons [52] using mitochondrial modulators. Mitochondrial respiration is basal respiration that is sensitive to the combination of oligomycin (ATP synthase inhibitor), rotenone (Complex I inhibitor) and antimycin (Complex III inhibitor). Mitochondrial respiration was lower in A53T neurons than in wt neurons (t-test, p < 0.05) at 7 DIV (Figure 5C and 5D). Mitochondrial respiration that is sensitive to oligomycin is ATP-linked respiration. ATP-linked respiration was lower in A53T neurons than in wt neurons (t-test, p < 0.05) at 7DIV (Figure 5C and 5D). Mitochondrial respiration that is insensitive to oligomycin results from proton leak. The proton leak in A53T neurons and wt neurons was similar (t-test, p > 0.05) at 7 DIV (Figure 5C and 5D). Non-mitochondrial respiration is basal respiration that is insensitive to the combination of oligomycin, rotenone and antimycin. Non-mitochondrial respiration was higher in A53T neurons than in wt neurons (t-test, p < 0.05) at 7 DIV (Figure 5C and 5D). The maximum respiratory capacity is the sum of the respiration that occurs upon sequential addition of oligomycin and FCCP (mitochondrial uncoupler) and the respiration that results in proton leak. The A53T neurons showed a lower maximum respiratory capacity than the wt neurons (t-test, p < 0.05) at 7 DIV (Figure 5C and 5D). Neurons at 13-14DIV exhibited a similar profile (Figure 5E) to that seen with neurons at 7 DIV (Figure 5D). Overall the results suggested that A53T α-syn impairs mitochondrial respiratory function.

### A53T-induced mobility changes are reversible

**Figure 6:**
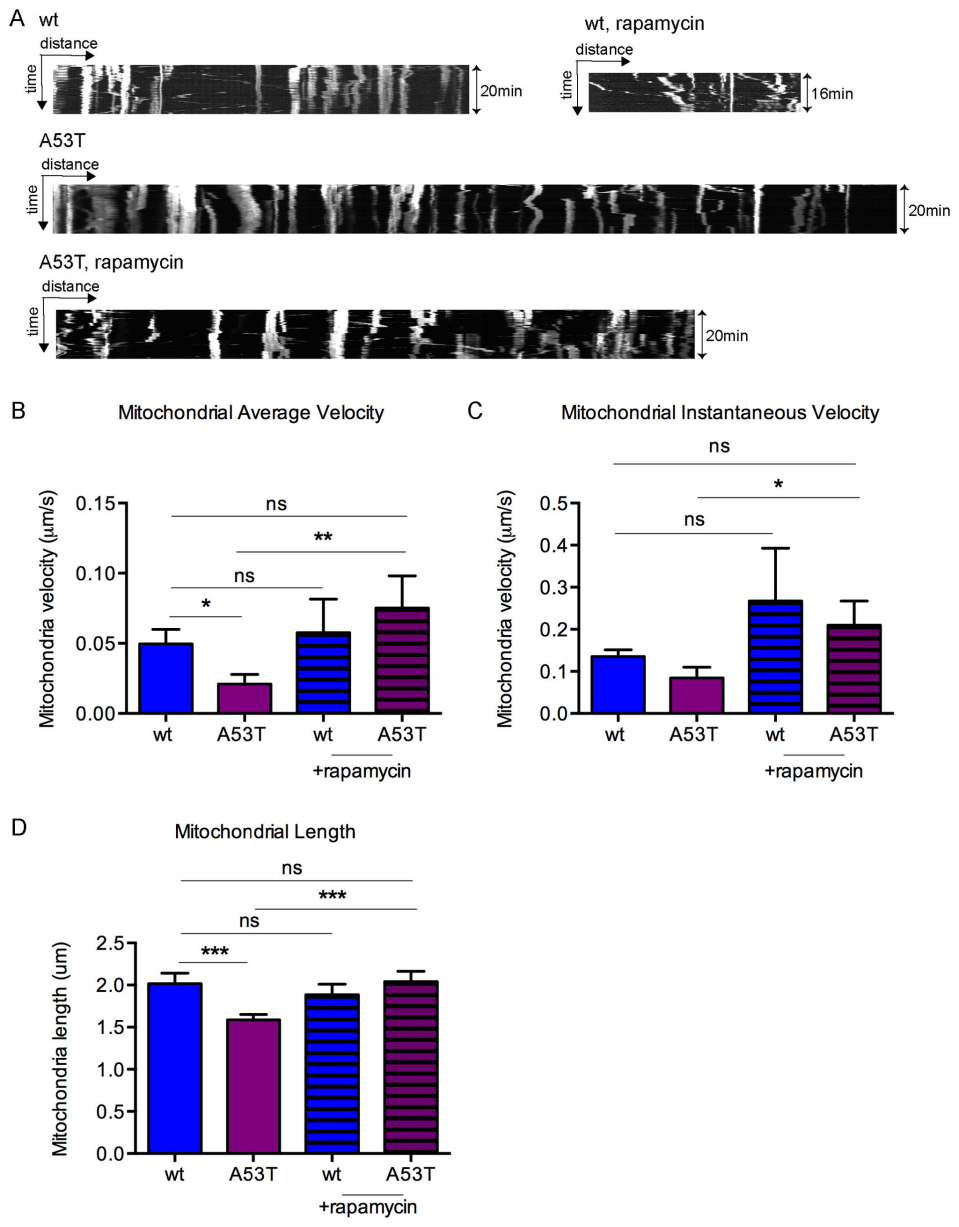
Rapamycin rescued mitochondrial mobility in A53T α-syn neurons. **A**, Representative kymographs showing treatment of rapamycin (1μM) for 7-9hr increased mobility of mitochondria in A53T α-syn neurons (7DIV). Images were acquired at the interval of 12s for 20min. B, C, Rapamycin rescued mitochondrial transport in A53T neurons to the level seen in wild-type cultures. One representative experiment is shown and 58-130 mitochondria were tracked in each condition. Experiments were repeated 3 times and results were consistent across all experiments. **D**, Rapamycin also rescued mitochondrial length in A53T neurons (6-7DIV) to the level seen in wild-type cultures. The results were pooled from three experiments. Mean +/- SEM is shown and t test was used to compare velocity in different conditions. *p<0.05; **p<0.01; ***p<0.001; ns=non-significant. The error bars depict SEM. wt=wild type, A53T=A53T α-synuclein, μm=micrometers, s=seconds, min=minutes.

Previous studies showed mutant α-syn impaired chaperone-mediated autophagy (CMA) [53,54] and wild-type α-syn inhibited macroautophagy in cells [55]. We hypothesized that mitochondrial mobility can be reversed by autophagy induction. Rapamycin is a compound with many potential beneficial mechanisms of action in the context of PD models, including enhanced autophagy, reduced translation, activation of 4E-BP and anti-apoptotic activity [56]. In order to evaluate whether A53T-induced changes in mitochondrial mobility are reversible, we treated primary neurons with rapamycin. We investigated the effect of rapamycin on mitochondria mobility in A53T α-syn neurons. At 7DIV, wt or A53T α-syn neurons transfected with DsRed-mito were incubated with rapamycin (1μM) for 7-9 hr. Live confocal time-lapse images were acquired and mitochondrial transport was recorded (Movies S1-S3). Consistently, the majority of mitochondria were stationary in A53T α-syn neurons (Figure 6A; Movie S2) and the average velocity of mitochondrial transport in A53T α-syn axons (0.021±0.007μm/s, n=130) was reduced significantly compared to that in wt axons (0.050±0.010μm/s, n=58, Movie S1) (Figure 6B). More mitochondria were mobile in A53T neurons with the treatment of rapamycin (Movie S3). In the presence of rapamycin, mitochondrial mobility did not change significantly in wt neurons, however, the average velocity in A53T neurons increased to 0.075±0.023μm/s (Figure 6B). There was no difference between mitochondrial mobility in wt neurons vs. that in A53 neurons treated with rapamycin (Figure 6B and 6C). Thus, rapamycin rescued mitochondrial mobility in A53T neurons, demonstrating full reversibility of this phenotype. In addition to the effects on mitochondrial mobility, rapamycin was also able to rescue the A53T α-syn mediated effects on mitochondrial length (Figure 6D) and mitochondrial membrane potential in A53T neurons (Figure S5). The A53T α-syn mediated effects on mitochondrial length and MitoTracker Red-measured membrane potential were more subtle than the mitochondrial mobility, but were significant and, more importantly, fully reversed by rapamycin treatment. 

## Discussion

Our results in cortical neuronal cultures from mice expressing human A53T α-syn reveal several novel effects of A53T α-syn on mitochondrial biology including trafficking and bioenergetics, and also a potential mechanism of rescuing the mitochondrial transport defect via autophagy induction and removal of α-syn. First, mitochondrial mobility was significantly reduced in A53T α-syn cortical neurons compared to that in wild-type neurons. Second, we show that trafficking of autophagosomes indicated by EGFP-LC3-positive puncta significantly increased in A53T α-syn neurons. In addition, transport of cargo carrying the synaptic vesicle protein synaptophysin was not affected. Taken together, these data suggest that A53T α-syn selectively inhibited mitochondrial mobility rather than inhibiting general cargo trafficking. However, A53T α-syn did not significantly affect mitochondrial morphology except for mitochondrial length at 6-7DIV. Third, mitochondrial membrane potential (ψ_m_) was reduced in A53T α-syn neurons and mitochondrial respiration as well as the maximum respiratory capacity were reduced. Finally we demonstrate rapamycin reversed the deficits in mitochondrial mobility, length and membrane potential observed in the A53T α-syn primary neuronal cultures.

Previous studies have linked mitochondrial defects with neurodegenerative diseases [14] and emerging evidence has shown that mitochondrial trafficking is impaired in multiple models of neurodegenerative diseases including Parkinson’s, Alzheimer’s and Huntington’s disease [15,50,57-59]. For example, MPP+, which can induce Parkinson-like symptoms in primates, was found to rapidly reduce mitochondrial trafficking in neurons [50]. However, it was unknown if mutant α-syn (A53T) affects mitochondrial transport. In this study, we found that the average velocity of mitochondrial transport was reduced by ≥85% in cortical neurons from mice expressing human A53T α-syn at both 6-7DIV and 14DIV. Interestingly the mitochondrial velocity was much slower at 6-7DIV compared to that at 14DIV. It is possible that synaptogenesis increased mitochondrial trafficking. Together with the MPP+ study, it suggests that defects of mitochondrial mobility may contribute to pathology of PD. During the course of our work, a paper was published [21] which demonstrated that mitochondria transport was reduced in primary hippocampal neurons from transgenic mice that expressed both A53T α-syn and mito-CFP. In general our observations are consistent with theirs, and we extend this work to show that the mitochondrial transport deficits are reversed with the rapamycin treatment.

In order to evaluate the specificity of the A53T α-syn associated mitochondrial mobility deficit, we also investigated movement of other cargo including autophagosomes (GFP-LC3) and the synaptic vesicle protein synaptophysin. Neither of them were significantly inhibited in A53T α-syn neurons. Overall the results suggested A53T α-syn specifically impaired mitochondrial movement. In addition, previous results [21] have shown that overexpression of β-syn did not inhibit mitochondrial mobility in neurons, thus ruling out the possibility that the effect overexpression. Interestingly, the velocity of autophagosome movement increased significantly in A53T α-syn neurons, suggesting autophagy is affected in A53T α-syn neurons to some extent (Berger et al., unpublished data). Autophagy is a multi-step process including induction, formation and transport of autophagosomes, fusion of autophagosomes with lysosomes and degradation [40], so the precise mechanisms are likely complex but previous studies have shown that mutant α-syn impaired chaperone-mediated autophagy (CMA) [53,54] and wild-type α-syn inhibited macroautophagy in cells [55]. Aggregate-prone proteins can be degraded through autophagy [60,61]. Up-regulation of autophagy has become an attractive therapeutic strategy in neurodegenerative diseases with a common feature of aggregated proteins. Rapamycin, a US Food and Drug Administration (FDA)-approved antibiotic and immunosuppressant drug, inhibits the activity of mammalian target of rapamycin (mTOR) and it has been shown that rapamycin induces autophagy in cell lines, primary neurons and animals [62-66]. In this study, we showed that rapamycin reversed the mitochondrial mobility deficits in the A53T α-syn primary cortical neurons. Since we also demonstrated that rapamycin can up-regulate autophagic flux in primary cortical neurons (Berger et al., unpublished data), it is likely that rapamycin reduced levels of toxic A53T α-syn through autophagy induction. We do observe a subtle (approximately 10%) reduction in total α-syn levels in rapamycin treated cultures (Berger et al., unpublished data). However, this does not preclude the possibility that induction of autophagy results in higher clearance of low levels of toxic α-syn species (e.g. oligomers / small aggregates) that are below the limit of detection by the immunoblot methods we have used, especially in a system where α-syn is constitutively expressed under a strong promoter. Overall our studies suggest that autophagy induction can benefit neurons by reducing toxic A53T α-syn species (oligomeric / aggregated) and rescuing mitochondria health, which may be a more-sensitive and physiological read-out of A53T α-syn-induced neurotoxicity. It supports the hypothesis that autophagy induction and/or restoration of normal mitochondrial function could be a potential therapeutic treatment strategy for Parkinson’s disease.

Currently we do not know how A53T α-syn inhibits mitochondrial mobility. It has been shown that miro1, a motor adapter protein, is required for mitochondrial transport along axons [67]. More recently two publications demonstrated that PINK and Parkin down-regulate levels of miro1 to stop mitochondrial mobility [68,69]. Thus one possibility is that expression of A53T α-syn decreases the levels of miro1 and eventually inhibits mitochondrial transport. We tested this possibility by probing miro1 levels with anti-miro1 antibodies in Western blots, but there was no difference of miro1 levels in wt vs. A53T α-syn neurons (unpublished observations). A second possibility is that A53T α-syn increases Ca^2+^ signal in neurons since increased Ca^2+^ influx was shown to inhibit mitochondrial movement [25,70]. Supporting this potential mechanism are previous studies suggesting that A53T α-syn can form Ca^2+^ permeable pores in the plasma membrane [71] and it can regulate Ca^2+^ entry pathways [72]. We showed that A53T α-syn not only reduced overall mitochondrial mobility but it also reduced the percentage of mobile mitochondria. In other words, the percentage of stationary mitochondria increased in A53T α-syn neurons. Thus it is also possible that A53T α-syn regulates syntaphilin or myosin [32,73] to enhance anchoring of stationary mitochondria.

Recombinant wild type and mutant human α-syn have been shown to depolarize dissociated mitochondria *in vitro* [74] and overexpression of wt and mutant human α-syn induced loss of ψ_m_ in SH-SY5Y cells [19]. Loss of ψ_m_ was also found in MPP+ treated neurons [50], skin cells from Parkinson patients with LRRK2 G2019S mutation [75] and cells with deficiency of DJ-1, a gene identified in early-onset recessive parkinsonism [76]. Interestingly mutant huntingtin, which is the cause of Huntington’s disease, was also shown to reduce ψ_m_ [77] suggesting mitochondria depolarization may be a common pathological hallmark in chronic neurodegenerative diseases. We demonstrate that Δψ_m_ was reduced in primary cortical neurons expressing A53T α-syn by using two Δψ_m_-sensitive dyes (MitoTracker red and TMRM). With reduced Δψ_m,_ mitochondria in A53T α-syn are expected to consume less oxygen. Previous reports have shown that brain mitochondria [78] and dopaminergic synaptosomes [18] from human A53T α-synuclein mice show a lower Complex I activity than that from age-matched wt controls. Our data showed that the A53T neurons showed a lower OCR/ECAR ratio and a lower maximum respiratory capacity than that of wt neurons. To the best of our knowledge, this is the first report to show that these endpoints are reduced in neurons from human A53T α-synuclein mice. Overall results suggest that loss of Δψ_m_ may represent an important pathological change in Parkinson’s disease. Targeting mitochondrial function, especially Δψ_m_, either directly, e.g. via mitochondrial biogenesis, or indirectly, e.g. by stimulating autophagy and clearance of the toxic proteins and damaged mitochondria, may be a novel therapeutic strategy for the treatment of Parkinson’s disease.

## Supporting Information

Figure S1 **Expression of human A53T α-syn in cortical neurons.** Representative fields from A53T (left) and wt (right) cultures at 7DIV. Dendrites were labeled with anti-MAP2 antibodies (green) and human A53T α-syn (red) was detected with antibodies specific to human α-syn (Covance 4B12). Nuclei were counterstained with DAPI (blue).
(JPG)

Figure S2 **Transport velocity of synaptic vesicles was not inhibited in A53T α-syn neurons.** **A**,**B**, Representative kymographs depicting movement of synaptic vesicles labeled by mCherry-synaptophysin. **C**, Bar graphs showing that velocity of synaptic vesicles was not significantly reduced in A53T neurons. Mean +/- SEM is shown and t test was used (n.s.=non-significant). wt=wild type, A53T=A53T α-synuclein, μm=micrometers, min=minutes.
(JPG)

Figure S3 **Mitochondrial length/width at 6-7DIV and 13-14DIV.** **A**, **B**, Summary of 3 independent experiments measuring mitochondria length and width at 6-7DIV. **C**, Summary of 3 independent experiments measuring mitochondrial length at 13-14DIV. Data plotted as Mean +/- SEM. t-test (**p<0.01; ****p<0.0001). wt=wild type, A53T=A53T α-synuclein, DIV=days *in* *vitro*.
(JPG)

Figure S4 **A53T α-syn induced loss of mitochondrial membrane potential indicated by TMRM.** **A**,**B**, Representative images of mitochondria stained with TMRM. **C**, Bar graphs show a significant reduction in TMRM fluorescence intensity (normalized) in A53T neurons compared to wt neurons in 6DIV and 14DIV. Since accumulation of TMRM is dependent on mitochondrial transmembrane potential, it suggests a loss of mitochondrial membrane potential in A53T neurons. Data plotted as Mean +/- SEM. t-test (*p<0.05; ***p<0.001). Scale bar=20μm. wt=wild type, A53T=A53T α-synuclein, DIV=days *in* *vitro*.
(JPG)

Figure S5 **Rapamycin rescued mitochondrial membrane potential at 6DIV.** Bar graphs demonstrating A53T reduced mitochondrial membrane potential (MMP) indicated by MitoTracker Red intensity. Note the Y-axis starts from 80% and the difference between wt and A53T is subtle but highly significant. Rapamycin rescued the reduced MMP in A53T without affecting MMP in wt neurons. Data plotted as Mean +/- SEM. t-test (***p<0.001; ****p<0.0001). wt=wild type, A53T=A53T α-synuclein.
(JPG)

Table S1 **Parameters of mitochondrial shape at 6-7DIV.**
(XLSX)

Table S2 **Parameters of mitochondrial shape at 13-14DIV.**
(XLSX)

Movie S1 **Mitochondrial transport along the axon in the wt culture.** 101 frames from an image stack are played at the speed of 20 fps (frames per second). Imaging interval = 8 seconds.
(AVI)

Movie S2 **Mitochondrial transport along the axon in the A53T culture.** 101 frames from an image stack are played at the speed of 20 fps (frames per second). Imaging interval = 8 seconds.
(AVI)

Movie S3 **Mitochondrial transport along the axon in the A53T culture treated with rapamycin.** 101 frames from an image stack are played at the speed of 20 fps (frames per second). Imaging interval = 8 seconds.
(AVI)
