# Supplementary figures and images for: Human A53T α-Synuclein Causes Reversible Deficits in Mitochondrial Function and Dynamics in Primary Mouse Cortical Neurons
Source: PLoS One. 2013 Dec 31;8(12):e85815. doi: 10.1371/journal.pone.0085815 (PMC3877382; doi:10.1371/journal.pone.0085815)

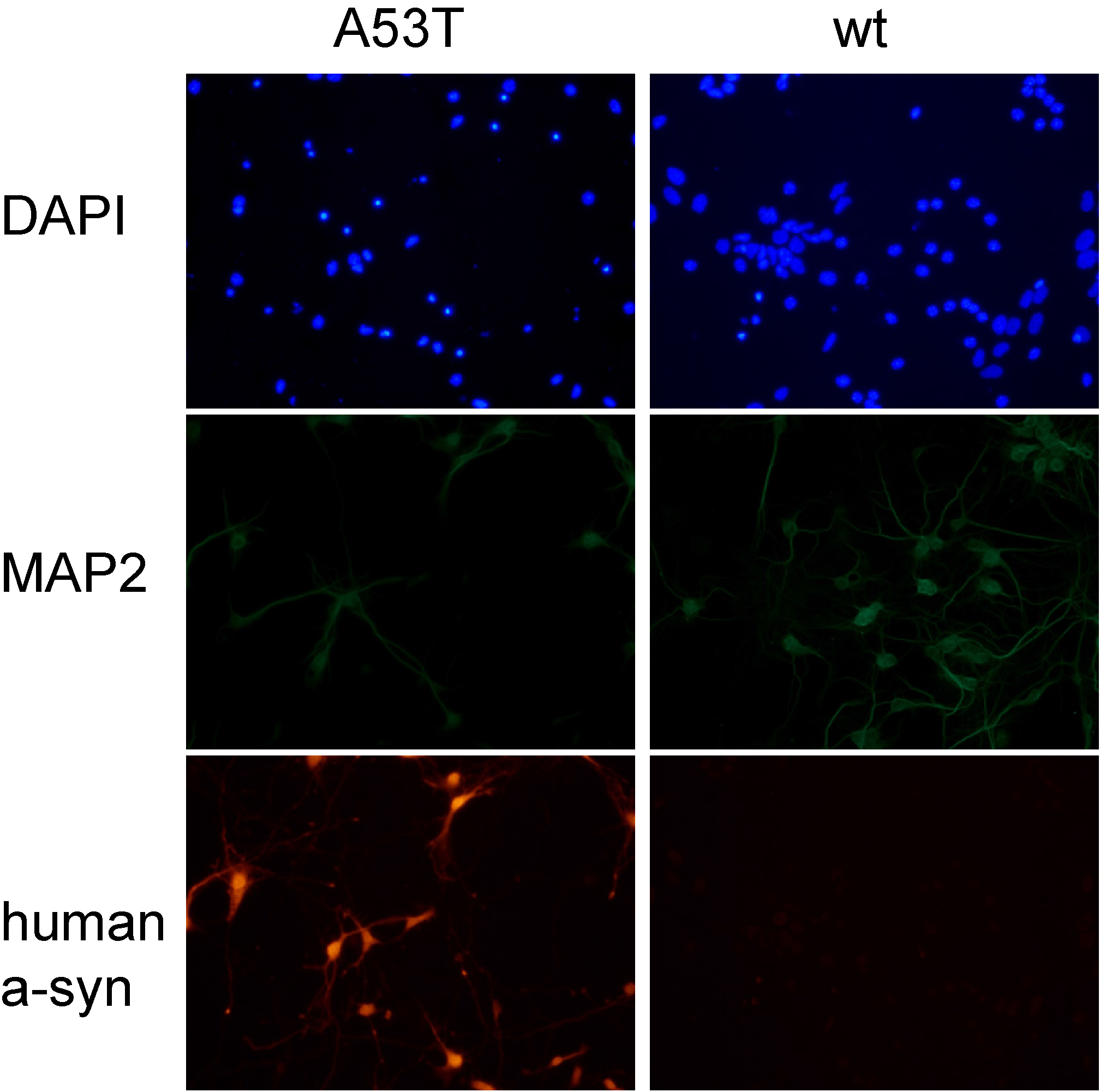

Supplement: Figure S1 — Expression of human A53T α-syn in cortical neurons. Representative fields from A53T (left) and wt (right) cultures at 7DIV. Dendrites were labeled with anti-MAP2 antibodies (green) and human A53T α-syn (red) was detected with antibodies specific to human α-syn (Covance 4B12). Nuclei were counterstained with DAPI (blue). (JPG) [file pone.0085815.s001.jpg]

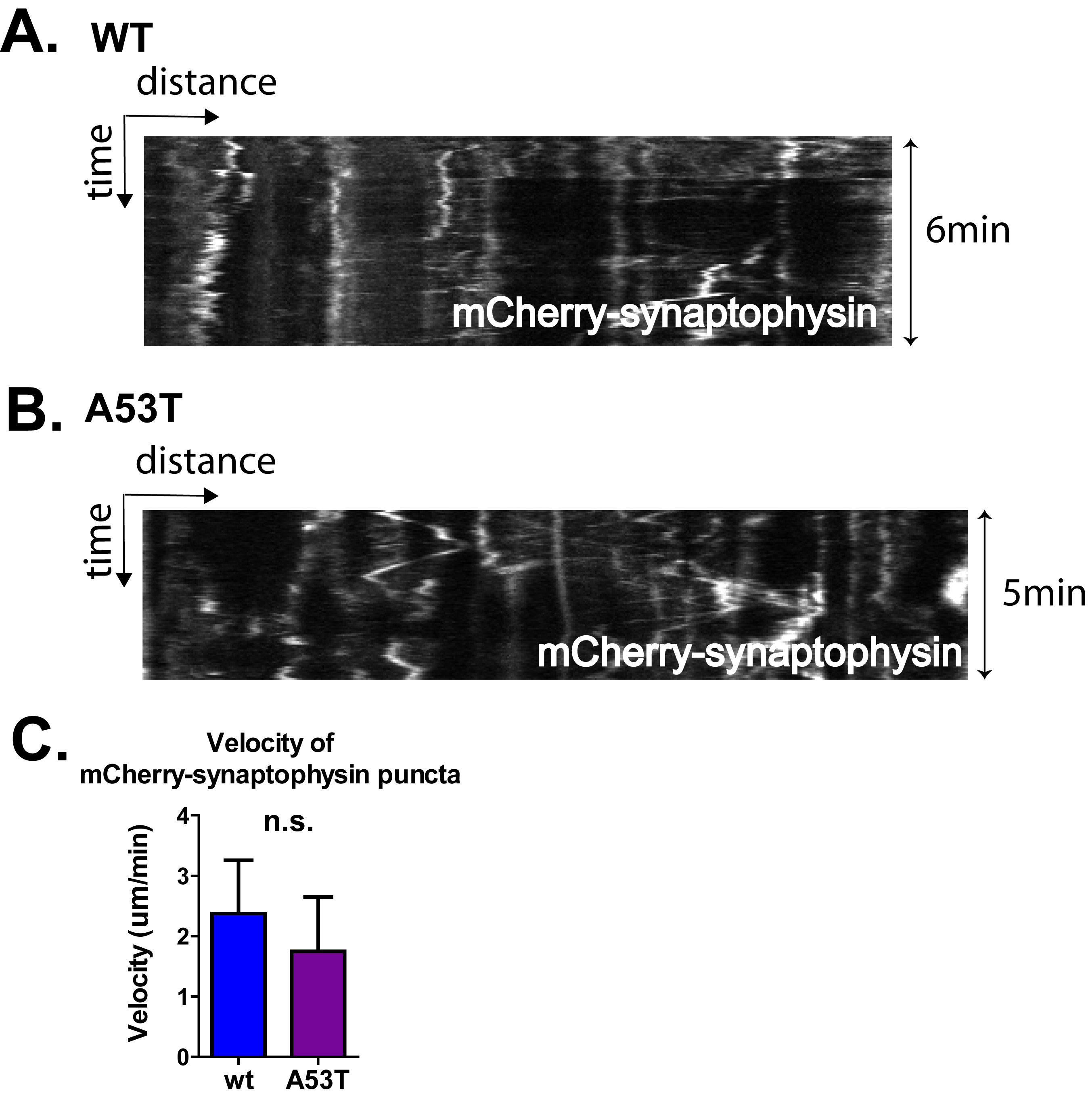

Supplement: Figure S2 — Transport velocity of synaptic vesicles was not inhibited in A53T α-syn neurons. A,B, Representative kymographs depicting movement of synaptic vesicles labeled by mCherry-synaptophysin. C, Bar graphs showing that velocity of synaptic vesicles was not significantly reduced in A53T neurons. Mean +/- SEM is shown and t test was used (n.s.=non-significant). wt=wild type, A53T=A53T α-synuclein, μm=micrometers, min=minutes. (JPG) [file pone.0085815.s002.jpg]

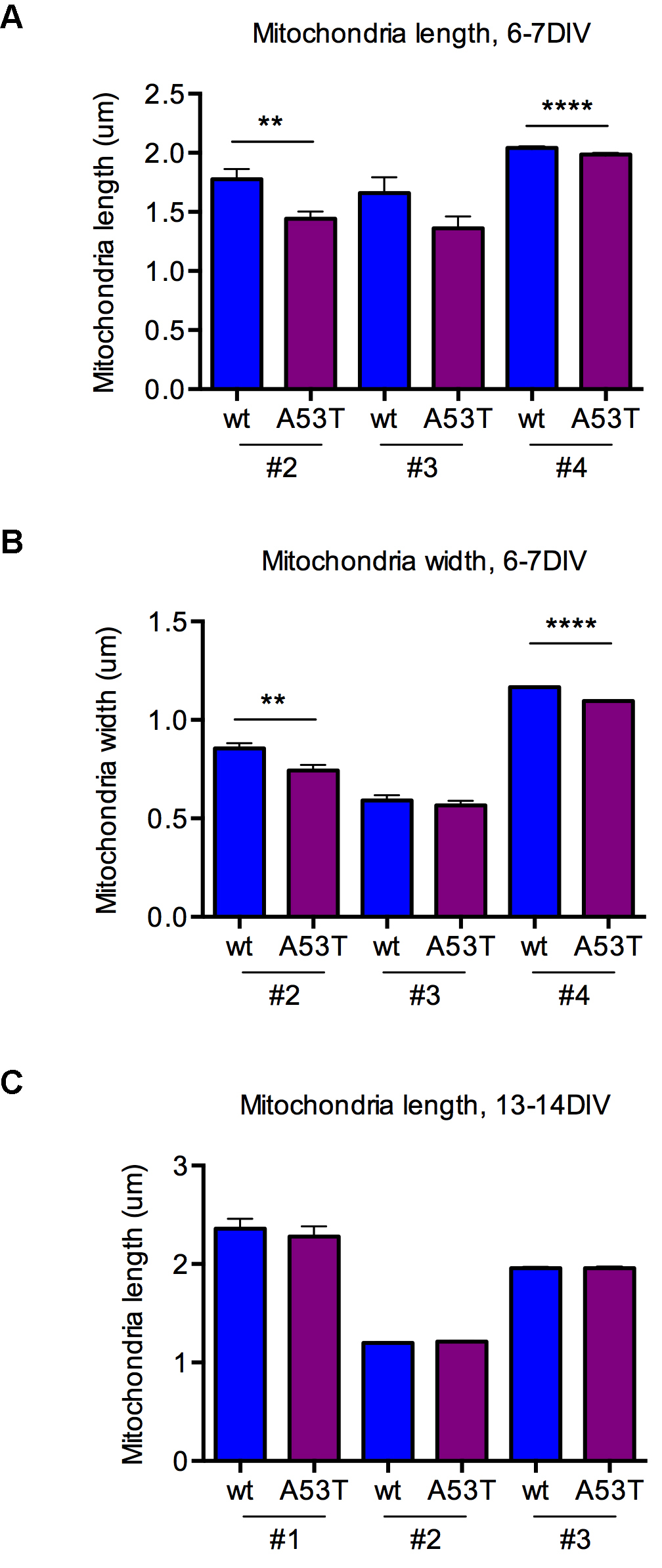

Supplement: Figure S3 — Mitochondrial length/width at 6-7DIV and 13-14DIV. A, B, Summary of 3 independent experiments measuring mitochondria length and width at 6-7DIV. C, Summary of 3 independent experiments measuring mitochondrial length at 13-14DIV. Data plotted as Mean +/- SEM. t-test (**p<0.01; ****p<0.0001). wt=wild type, A53T=A53T α-synuclein, DIV=days in vitro. (JPG) [file pone.0085815.s003.jpg]

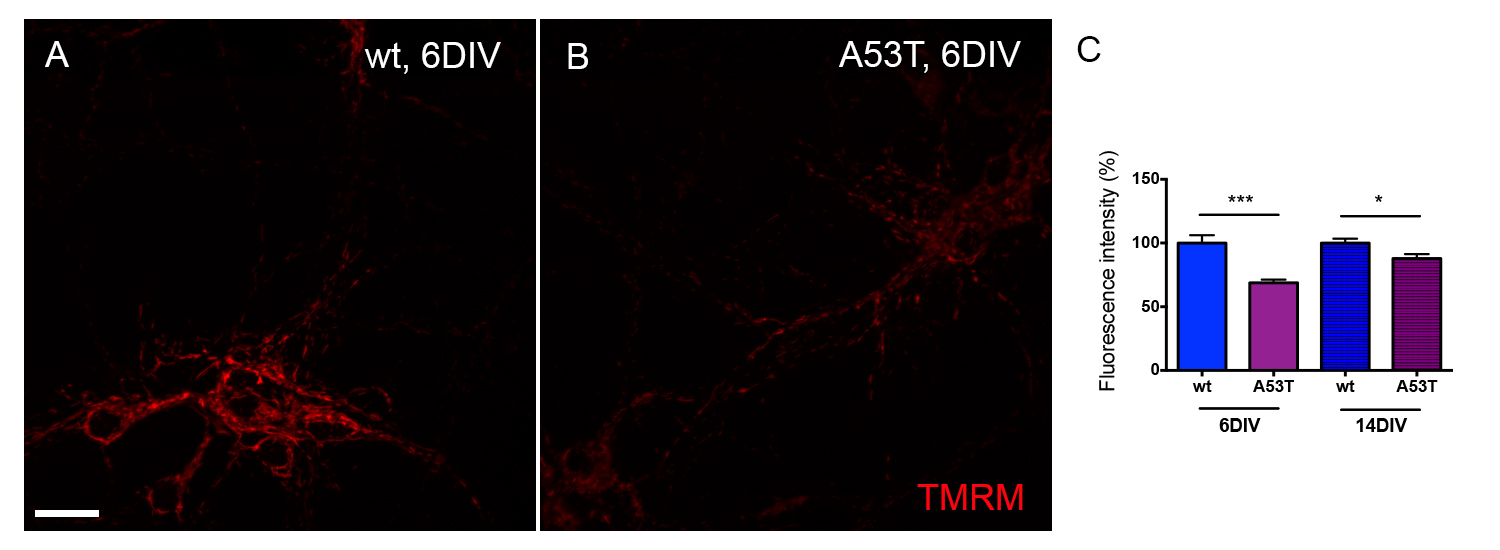

Supplement: Figure S4 — A53T α-syn induced loss of mitochondrial membrane potential indicated by TMRM. A,B, Representative images of mitochondria stained with TMRM. C, Bar graphs show a significant reduction in TMRM fluorescence intensity (normalized) in A53T neurons compared to wt neurons in 6DIV and 14DIV. Since accumulation of TMRM is dependent on mitochondrial transmembrane potential, it suggests a loss of mitochondrial membrane potential in A53T neurons. Data plotted as Mean +/- SEM. t-test (*p<0.05; ***p<0.001). Scale bar=20μm. wt=wild type, A53T=A53T α-synuclein, DIV=days in vitro. (JPG) [file pone.0085815.s004.jpg]

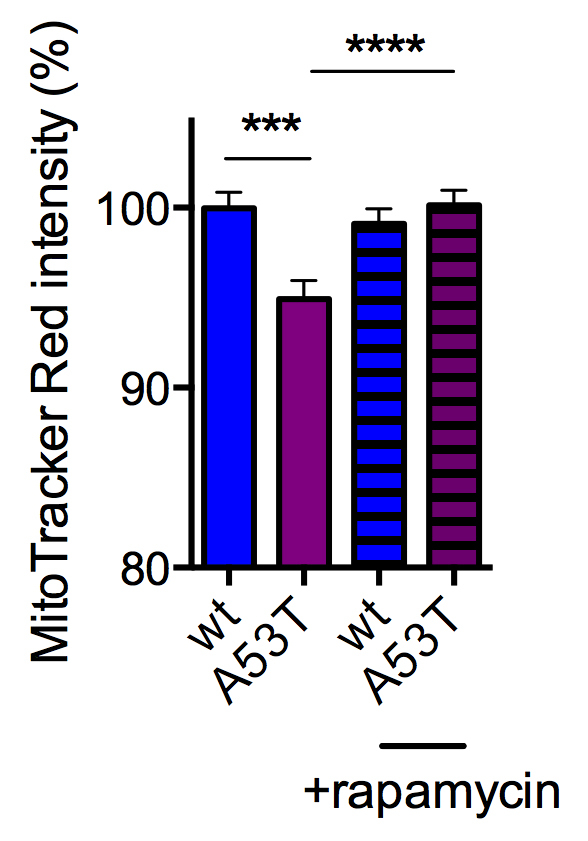

Supplement: Figure S5 — Rapamycin rescued mitochondrial membrane potential at 6DIV. Bar graphs demonstrating A53T reduced mitochondrial membrane potential (MMP) indicated by MitoTracker Red intensity. Note the Y-axis starts from 80% and the difference between wt and A53T is subtle but highly significant. Rapamycin rescued the reduced MMP in A53T without affecting MMP in wt neurons. Data plotted as Mean +/- SEM. t-test (***p<0.001; ****p<0.0001). wt=wild type, A53T=A53T α-synuclein. (JPG) [file pone.0085815.s005.jpg]
